# The stiffness of the extracellular matrix is a key factor in tumor progression

**DOI:** 10.1002/mco2.729

**Published:** 2024-09-07

**Authors:** Yue Zheng, Xinming Su, Shiwei Duan

**Affiliations:** ^1^ Department of Clinical Medicine Hangzhou City University Hangzhou Zhejiang China; ^2^ Shanghai Key Laboratory of Orthopaedic Implant, Department of Orthopaedic Surgery Shanghai Ninth People's Hospital Affiliated shanghai jiao Tong University School of Medicine Shanghai China; ^3^ Key Laboratory of Novel Targets and Drug Study for Neural Repair of Zhejiang Province, School of Medicine Hangzhou City University Hangzhou Zhejiang China

1

In a recent publication in *Nature* by Bansaccal et al., the significance of the extracellular matrix's (ECM's) structural framework in dermal invasion and tumor formation was underscored.^1^ Investigations revealed that skin with elevated density and tissue elasticity of type I collagen in the dermis effectively inhibits cell reprogramming induced by SmoM2 mutants, thus acting as a natural barrier against basal cell carcinoma (BCC) occurrence and migration (Figure 1).^1^

**FIGURE 1:**
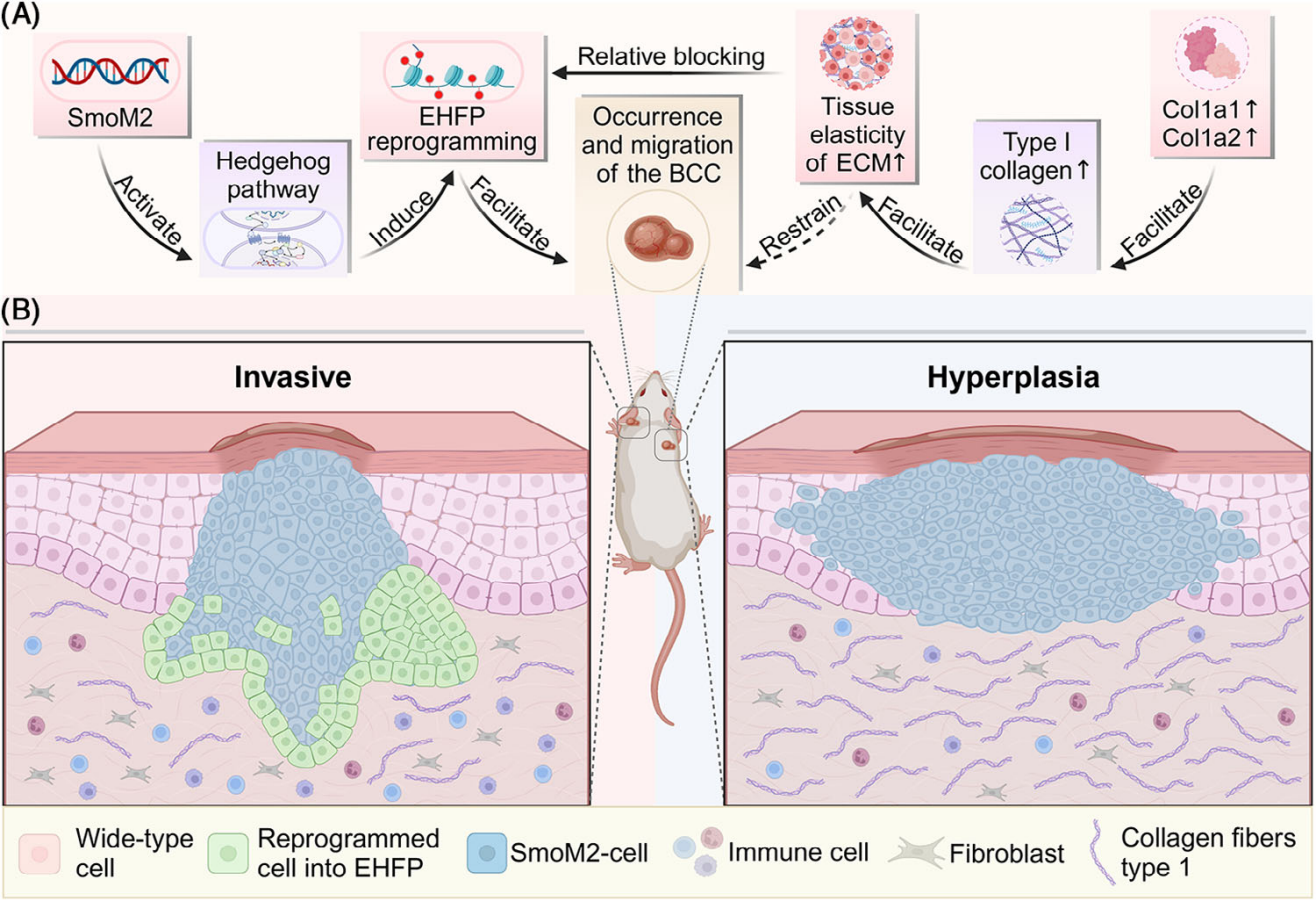
Regional variations in extracellular matrix (ECM) stiffness shape basal cell carcinoma (BCC) behavior. (A) Activation of the oncogene SmoM2 triggers the Hedgehog signaling pathway, leading to the genesis of epidermal BCCs and compromising the normal differentiation capacity of basal cells. This process entails the manifestation of characteristics akin to epidermal hyperplasia, fibrosis, and papillomatosis (EHFP) during dedifferentiation. Key genes encoding type I collagen, namely, Col1a1 and Col1a2, play pivotal roles in this cascade. Alterations in their expression levels directly impact the composition of type I collagen, thereby modulating the stiffness of the ECM. Given the regional disparities in ECM stiffness, the behavior of BCC cells exhibits marked divergence across different anatomical regions. (B) Within mouse ear skin, the presence of scant type I collagen fibers results in the formation of a sparse collagen fiber network. This architectural configuration facilitates facile invasion of BCC cells into the dermis, culminating in the formation of branch‐like structures. In contrast, the epidermal collagen fiber network in back skin exhibits higher density, presenting impediments to the penetration of BCC cells into deeper tissues. Consequently, BCC cells tend to adopt a predominantly horizontal tissue structure within back skin.

BCC, a prevalent nonmelanocytic skin cancer, originates from stem cells in the basal layer of the epidermis and hair follicles.^2^ Ultraviolet (UV) exposure is one of the most destructive environmental risk factors, particularly for individuals with light skin, a history of frequent sunburns, and older age, significantly increasing the incidence of BCC.^2^ More worryingly, patients with certain hereditary skin diseases, such as xeroderma pigmentosum and albinism, face an even higher risk of developing BCC.^2^ Notably, abnormal changes in tumor suppressor genes and proto‐oncogenes, such as the overactivation of the Hedgehog (HH) protein family and mutations in the tumor suppressor gene TP53, have been identified as key factors in promoting BCC formation.^2^

The ECM plays a pivotal role in regulating tissue development and homeostasis.^3^ It is composed of various macromolecules with distinct biochemical and biomechanical properties, including glycoproteins, collagens, and proteoglycans, which intertwine to form a complex three‐dimensional supramolecular network.^3^ This network structure finely regulates cell growth, survival, migration, and differentiation.^3^ In the tumor microenvironment, the ECM occupies a central position, profoundly affecting numerous characteristics of cancer and influencing all cellular processes involved in cancer occurrence, progression, and metastasis.^3^ Notably, the degradation of the ECM surrounding the tumor directly impacts the tumor invasion process.^3^ This degradation is accompanied by the high‐density and high‐tissue elasticity deposition of tumor‐specific ECM. Specifically, the ECM's structural integrity decisively impacts the physical properties of the TME (tumor microenvironment), notably its stiffness.^3^ However, our understanding of how different ECM regions regulate tumor behavior, particularly at the molecular level, remains incomplete. In‐depth research on the role of the ECM in BCC not only helps reveal the key regulatory mechanisms of the tumor microenvironment on tumor behavior but also provides a solid theoretical foundation for developing more precise and effective treatment strategies.

First, it is essential to recognize the significant regional specificity of the oncogenic mutant gene SmoM2 in tumor development in mice. To delve deeper into this phenomenon, the research team employed fluorescent labeling of SmoM2 mutants combined with intravital multiphoton confocal microscopy to conduct time‐dependent in vivo imaging in anesthetized mice. Significantly differing malignant behaviors were observed between BCC cells in the ear and those in the back. Ear BCC cells exhibited pronounced morphological changes, forming placode‐like structures and establishing high‐tension interfaces with surrounding wild‐type cells, leading to rapid transformation from lateral to vertical invasion with branch‐like structures penetrating the dermis. Conversely, dorsal BCC cells maintained their original morphology, lacking specific mechanical interfaces, and exhibiting only lateral expansion without vertical dermal layer invasion. To unravel the molecular mechanism underlying this phenomenon, the research team conducted time series data analysis of single‐cell transcriptomes. Results demonstrated varying degrees of embryonic hair follicle progenitor (EHFP) reprogramming among BCC cells in different spatiotemporal regions, resulting in distinct cellular behaviors.

Addressing this specific phenomenon, the research team further explored compositional differences in the dermis of the ear and back skin. Unbiased quantitative proteomic analysis revealed significantly increased expression levels of Col1a1 and Col1a2 genes encoding type I collagen fibers in the back skin dermis compared to the ears. This disparity elucidated the abundance of thick collagen fibers in the back skin dermis contrasted with the sparse collagen fiber network in the ears, indicating a consistent difference before and after SmoM2 expression. The authors demonstrated that high levels of type I collagen can inhibit the development of BCC by treating mouse skin with collagenase, thereby affecting the tissue elasticity of the ECM. Additionally, they expanded the clinical relevance of this finding by studying the skin of aged and UV‐irradiated mice, observing similar phenomena. This broadens the scope and potential clinical applications of their discovery.

The significant role of the ECM in tumor development has been extensively validated. Collagen, the primary component of the ECM—including types I, II, and III—possesses varying physical properties and spatial distributions, which influence tumor development by altering the tumor microenvironment and affecting cancer cell dormancy. At the same time, other properties of the ECM also play a crucial role in tumor occurrence and development, indicating that these areas may become promising research directions in the future. Recent studies have revealed that factors such as the pore size, matrix degradation ability, and viscoelasticity of the ECM can significantly affect cell behavior.^4^, ^5^ Overall, in addition to genetic characteristics, the tissue elasticity and composition of the ECM can be considered important indicators of cancer progression and malignancy, providing valuable guidance for treatment planning. These findings merit further verification in more types of cancer, including nonskin tumors, and larger sample sizes. Additionally, specific interventions in the cancer cell microenvironment based on ECM properties—such as guiding the controllable expression and functional enhancement of collagen through artificial design, or modifying other components like the elasticity of blood vessels within the ECM—show great potential for future precision cancer treatment. Therefore, these areas warrant more in‐depth and systematic research by scientific investigators. The authors verified the impact of ECM stiffness on tumor development through in vivo experiments. However, many researchers now construct in vitro models to simulate the tumor microenvironment, aligning with the 3R principles (Reduce, Reuse, and Recycle) of animal experimentation. In vitro models can also mimic the composition and structural properties of the ECM. Further development of these models can allow for the precise tuning of ECM stiffness, miniaturization, automation, high‐throughput drug screening, and in‐depth molecular research.

The study by Bansaccal et al. not only underscores the pivotal role of ECM physical properties in tumor development and progression but also offers profound insights into the intricate interplay among genetic mutations, tumor progression, and ECM dynamics. Precise diagnosis and treatment strategies targeting ECM are anticipated to invigorate the development of personalized treatment approaches, warranting further exploration and investigation.

## AUTHOR CONTRIBUTIONS

Yue Zheng and Xinming Su analyzed the literature, wrote the manuscript, and drafted Figure 1. Xinming Su and Shiwei Duan conceived the idea. Shiwei Duan reviewed and revised the manuscript. All authors gave the final approval of the submitted version. All the authors have read and approved the final manuscript.

## CONFLICT OF INTEREST STATEMENT

The authors declare no conflicts of interest.

## ETHICS STATEMENT

Not applicable.

## Data Availability

Not applicable.
